# Ion Channel-Target Toxicology

**DOI:** 10.1155/2012/501438

**Published:** 2012-09-06

**Authors:** Yonghua Ji, Maria Elena De Lima

**Affiliations:** ^1^Lab of Neuropharmacology and Neurotoxicology, Shanghai University, Nanchen Road 333, Shanghai 200444, China; ^2^Departamento de Bioquímica e Imunologia, Instituto de Ciências Biológicas, Universidade Federal de Minas Gerais, Avenida Antônio Carlos 6627, 31.270 901 Belo Horizonte, MG, Brazil

The saying “use poison as an antidote to poison” has long been considered the spirit of traditional Chinese medicine and herbalism since Ming Dynasty and has indicated the medical values of toxins arising from venomous animals, plants, microbes, or synthetic chemicals. A well-known physician Sir P. M. Latham (1789–1875) once defined the relationship between poisons and medicines as “oftentimes the same substances given with different intents”, what highlighted the double-edged sword nature of toxins when applied with different doses.

In view of the great potential of toxins as medicines and drug precursors, a stormy survey and pursuit has been implemented within pharmaceutical industry and research centers in a worldwide range. A classical example comes from the studies of a Brazilian scientist, Dr. Sergio Ferreira, who discovered, in the 1960s, bradykinin potentiating peptides (BPPs), extracted from the venom of the Brazilian viper *Bothrops jararaca*. These peptides were later shown to be able to decrease arterial pressure. This study was very important for the development of an analogue molecule, by Squibb Laboratory, called Captopril, one of the most successful examples of blockbuster drugs, used as an angiotensin converting enzyme inhibitor (ACEi), currently used in the treatment of hypertension, generating more than 8 billion dollars a year.

Nowadays, a representative instance comes from the successfully commercialized toxin product “Ziconotide,” developed by Eli Lilly Incorporated (USA), from the venom of *Conus*. On the other side, JZMed Incorporated, a worldwide renowned market research firm specialized in the Chinese pharmaceutical industry, once forecasted that the Chinese preclinical and toxicology outsourcing industry would likely grow in a compound annual growth rate of 27% in five years after 2010 and its market value would likely reach more than $760 million by 2015.

Despite the rapid growth and worldwide attention to toxin R&D industries, it has to be well recognized that the productivity of discovering a new medicine from toxins is still little efficient in the global biopharmaceutical industry. The hindrances toward toxin-drug transformation may include but not be limited to (1) overall costs of drug development; (2) elimination of nondevelopable drug candidates; (3) extensive toxicology and safety pharmacology researches for any drug candidates. Among them, one of the major challenges of toxicity evaluation and integrated efficacy may be the targets of toxins in the human body.

Up to now, it has been well documented that ion channels are specific targets for numerous toxins. Neurotoxins secreted by various venomous species have played critical roles in understanding the physiological contributions of ion channels to the neuronal network as well as in probing and correlating ion channel structure and function. Ion channel-targeted toxins have been validated as promising drug candidates or leading molecules for the treatment of various neuronal syndromes.

All the above considerations led us to gather the current state of the art on natural toxins or toxic compounds targeted to ion channels with possible applications in health care and biotechnology.

This special issue provides a timely focus on the recent progress in our understanding of the mechanisms of toxins specially involved in neuronal dysfunctions. Geographically dispersed excellent specialists in this area from China, Brazil, USA, and France contributed to this special issue with 6 papers: 5 reviews and 1 full research article, covering from structure-function relationship to the neurological diseases involved with ion channel-targeted toxins, including the study of novel animal models. Although this special issue is a first attempt in the field of ion channel-targeted toxicology, a subsequent and more expanded issue with complementary topics shall be released in the near future.

The development in the field of toxinology owes much to the use of various nervous preparations obtained from various animal models. Among them, the biophysical principles of the nervous system function in insects have been shown to be the same as in mammals. In both groups of animals, similar neurotransmitters can be found, although their distribution varies. M. Stankiewicz et al. present the role of the nervous system of the cockroach* Periplaneta americana* as a well-addressed preparation in the development of toxinological studies. In addition, they provide a systematic introduction to electrophysiological methods to be applied therewith, which allow us to perform pharmacological tests in various levels of the nervous system organization. They also reconsider the definite statement concerning the mode of action of any toxin when carrying out experiments under different conditions.

Considered fundamental in many physiological processes, K^+^-channels are recognized as potential therapeutic targets in the treatment of several central nervous system diseases. C. D. C. Gati et al. provide an overview of CNS K^+^-channels involved in memory acquisition and storage and evaluate the use of highly selective K^+^-channel blockers derived from arthropod venoms as potential therapeutic agents for CNS diseases involving learning and memory mechanisms. Particularly, M.-F. Martin-Eauclaire and P. E. Bougis summarized recent work on the molecular mechanism of toxin-channel interactions of several high-affinity blockers selective to various K^+^ channels (SK_Ca_, K_v_4.x, and K_v_1.x K^+^ channel families), from the venom of the Moroccan scorpion *Androctonus mauretanicus mauretanicus, *which may provide new insights into the targets and the mode of action of KTxs.

Even though the studies of ion channel structure-function relationship have always been the most pursued spots, the posttranslational modification processes, such as glycosylation, phosphorylation, and alternative splicing associated with channel functions, have also been investigated. Z. Liu et al. proposed that sodium channel-specific neurotoxic toxins, a family of long-chain polypeptides originating from venomous animals, potentially share binding sites adjacent to glycosylated regions of VGSCs (voltage gated sodium channels). Thus, an interaction between toxins and glycosylated VGSCs might hopefully join the campaign to approach the role of glycosylation in modulating VGSC-involved neuronal network activity.

Apart from voltage-gated ion channels, ionotropic glutamate receptors classified as ligand-gated ion channels, such as NMDA, AMPA, and kainate receptors, are also thought to mediate much of the excitatory neurotransmission in the brain. D. R. Morris and C. W. Levenson discussed the role of the excitotoxic influx and zinc accumulation, the mechanisms responsible for its cytotoxicity, and a number of disorders of the central nervous system linked to these neuronal ion channels and zinc toxicity. The in-depth researches in toxic chemicals targeted on neuronal receptors may hopefully help to develop strategies to block zinc-mediated damage and prevent undesirable outcomes.

A very interesting study was developed by L. C. Carrijo-Carvalho et al., who discovered a peptide believed to be involved in development, regeneration, and pathological processes. It is abundant in the venom of the caterpillar* Lonomia obliqua*. Notably, this research evaluated the effects of this peptide based on lipocalin motif in human fibroblasts in morphogenesis and tissue homeostasis. This was the first paper of lipocalins modulating fibroblasts and ECM proteins.

We extremely appreciated the great contributions of the researchers who have warmly participated in this special issue as writers or reviewers. It is our best wish that the special issue presented here incites new studies that provide a better understanding of the mechanisms underlying ion channel-targeted toxicology, eventually leading to more effective treatments with toxin-evoked epidemiology or other health care concerns.

*Yonghua Ji* *Yonghua Ji* *Maria Elena De Lima* *Maria Elena De Lima*

